# Association between serum homocysteine level and unexplained infertility in in vitro fertilization/intracytoplasmic sperm injection (IVF/ICSI): A retrospective, hospital‐based, case‐control study

**DOI:** 10.1002/jcla.23167

**Published:** 2019-12-25

**Authors:** Linli Liu, Zhou Lin, Peihong Lin, Zhongqing Jiang

**Affiliations:** ^1^ Department of Gynaecology and Obstetrics Fuzhou First Hospital Affiliated to Fujian Medical University Fuzhou China

**Keywords:** association study, homocysteine, in vitro fertilization/intracytoplasmic sperm injection, unexplained infertility

## Abstract

**Background:**

Lower serum homocysteine (Hcy) levels are found to correlate with a better chance of clinical pregnancy and better embryo grades in assisted reproductive technology (ART). However, there is little knowledge on the association between Hcy level and unexplained infertility until now.

**Methods:**

A total of 388 infertile women undergoing IVF/ICSI treatments were recruited, including 129 women with unexplained causes (case group) and 259 women with known causes (control group), and the case group was further divided into subgroups A (≤8 μmol/L), B (>8 and <15 μmol/L), and C (≥15 μmol/L) based on the serum Hcy level. The associations between serum Hcy level and IVF/ICSI pregnancy outcomes were examined in infertile women with unknown causes.

**Results:**

A significantly higher serum Hcy level was measured in the case group than in the control group (*P* = .008). Subgroup analysis revealed a significant difference in the total number of oocytes retrieved among subgroups A, B, and C (*P* = .031), and no significant difference was seen among these three groups in terms of age, BMI, E_2_ level on the hCG day, number of M‐II oocytes, number of fertilized oocytes, or total number of high‐quality embryos (*P* > .05). Spearman correlation analysis revealed a negative correlation between serum Hcy level and total number of oocytes retrieved (*r* = −.406, *P* = .019). Univariate and multivariate linear regression analyses revealed that serum Hcy level had no correlations with any IVF/ICSI outcomes.

**Conclusion:**

Serum Hcy level has no associations with IVF/ICSI pregnancy outcomes.

## 1 INTRODUCTION

Infertility, a disease of the reproductive system, is defined as the inability to conceive despite 12 months of unprotected sexual intercourse,^1^ and this disorder has become a worldwide public health issue.^2^ Globally, it is estimated that approximately 15% of all couples suffer from infertility,^3^ and results from the systematic analysis for the Global Burden of Disease Study 2015 showed that there were 113 million infertile individuals and 752.4 thousand years lived with disability (YLDs) worldwide in 2015.^4^ Unexplained infertility, a form of infertility that cannot be explained by anovulation, poor sperm quality, tubal pathology, or without any known cause,^5^ is one of the most common infertility diagnoses, and there are about 30% of global infertile couples diagnosed with unexplained infertility.^6^ Although the pathogenesis of unexplained infertility has been extensively investigated,^7^, ^8^, ^9^ the exact etiology of this disorder remains unclear until now.

Homocysteine (Hcy), a sulfur‐containing non‐essential amino acid resulting from the metabolism of the essential amino acid methionine, plays a vital role in cellular homeostasis in man.^10^ Previous studies have shown that Hcy concentration correlates with sperm, oocyte, and embryo qualities.^11^, ^12^ Among 269 infertile women at a mean age of 37 ± 4 years, there were 69% presenting adequate plasma Hcy levels,^13^ and the pregnant women complicated with preeclampsia had significantly higher serum Hcy levels than healthy pregnant women at the same gestational age (*P* < .001).^14^ In addition, Hcy is reported to be inversely associated with fertility outcome,^15^ and lower Hcy levels have been found to correlate with a better chance of clinical pregnancy and better embryo grades in assisted reproductive technology (ART).^16^, ^17^ However, there is little knowledge on the association between Hcy level and unexplained infertility. In this retrospective, hospital‐based, case‐control study, we aimed to examine the association of Hcy with unexplained infertility in in vitro fertilization/intracytoplasmic sperm injection (IVF/ICSI).

## 2 SUBJECTS AND METHODS

### 2.1 Study subjects and grouping

A total of 388 infertile women undergoing IVF/ICSI treatments at Fuzhou First Hospital Affiliated to Fujian Medical University (Fuzhou, China) during the period from January through December 2017 were recruited, including 129 primary infertile women with unexplained causes (case group) and 259 primary infertile women with known causes (control group). Of the 259 women with known causes of infertility, there were 80 women (30.9%) due to tubal abnormalities, 37 cases (14.3%) due to endometriosis, 60 cases (23.2%) due to male infertility, and 62 cases (23.9%) due to anovulatory cycles, including 20 women (7.7%) exhibiting more than one cause of infertility. All patients’ demographic and clinical characteristics were captured from the medical records.

We collected the patients’ medical history, detected the serum Hcy level on the 3rd day of menstruation, and performed standard fertility testing, including karyotype analysis, semen analysis, cycle monitoring, hormonal profile on the 3rd day of menstruation, anti‐Müllerian hormone (AMH), uterine cavity as documented by transvaginal ultrasonography, and tubal patency as documented by hysterosalpingography (HSG) or laparoscopy with dye test, to investigate the causes of infertility before ART cycle. Unexplained infertility was diagnosed when the standard infertility evaluation results were normal.^18^

The 129 women with unexplained causes were then further divided into subgroups A (serum Hcy level of 8 μmol/L or less), B (serum Hcy level > 8 μmol/L but < 15 μmol/L), and C (serum Hcy level of 15 μmol/L or greater) according to the serum Hcy level.

### 2.2 IVF/ICSI procedure

In the standard long protocol, a gonadotropin‐releasing hormone agonist (GnRH‐a) leuprolide acetate (FamarL’Aigle) was given at a daily dose of 0.5 mg during the mid‐luteal phase of the previous cycle, followed by subcutaneous injection with recombinant human follicle‐stimulating hormone (FSH) Gonal‐F (Merck KGaA) at a daily dose of 150‐300 IU for the first six days. Subsequently, the daily dose of Gonal‐F was adjusted according to the follicular growth.

In the flexible antagonist protocol, Gonal‐F was administered at a daily dose of 150‐300 IU for the first six days, with a subsequent daily dose adjusted according to follicular growth. The GnRH antagonist cetrorelix acetate (Merck KGaA) was administered at a daily dose of 0.25 mg on the day when the mean diameter of the largest follicle was 14 mm.

In the prolonged protocol, pituitary downregulation was achieved by administration of a GnRH agonist triptorelin (IPSEN Pharma Biotech) at a standard full dose (3.75 mg) during the early follicular phase. The size and number of the antral follicles were monitored from day 1 or 2 of the menstrual cycle. A single intramuscular injection of GnRH‐a depot was given when the diameter of the antral follicles was <6 mm, and downregulation was confirmed after 28 days (no ovarian cysts > 8 mm and estradiol (E_2_) < 50 pg/L) before the gonadotropin stimulation started.

In the modified super‐long downregulation protocol, GnRH‐a (long‐acting diptorelin) 1.25 mg was injected intramuscularly once on day 2, and recombinant FSH was used 3 weeks post‐injection when E_2_ level was ≥20 and <50 pg/mL. GnRH‐a (short‐acting tiptorelin) 0.05 mg was administered every day when at least one of the dominant follicles was 10‐11 mm in diameter until the day human chorionic gonadotropin (hCG) was given.

During controlled hyperovulation (COH), participants were monitored closely with transvaginal ultrasound and serum hormone levels. When the E_2_ level was 600 pg/mL and greater, and there were at least 3 follicles with a diameter of 16 mm and higher, recombinant hCG (Ovidrel^®^ Prefilled Syringe; EMD Serono, Inc) was administered at a dose of 5000‐10 000 IU followed by oocyte retrieval 36 hours later. Embryo transfer (ET) was performed 3‐5 days after retrieval, and participants returned 14 days later for the measurement of serum hCG levels.

### 2.3 Ethical statement

This study was approved by the Ethics Review Committee of the Fuzhou First Hospital Affiliated to Fujian Medical University. Written informed consent was obtained from the participants following a detailed description of the purpose of the study. All experimental procedures were performed according to the Declaration of Helsinki.

### 2.4 Data analysis

Distribution of the data was analyzed with Kolmogorov‐Smirnov and Shapiro‐Wilk tests. All measurement data were expressed as mean ± standard deviation (SD), and categorical variables were described as number or proportion. Normally distributed data were compared between groups using independent‐samples *t* test, while non‐normally distributed data were compared with Mann‐Whitney *U* test. Differences of proportions were tested for statistical significance with chi‐squared test, and the statistical significance of differences among different group means was assessed by one‐way analysis of variance (ANOVA) with least significant difference (LSD) multiple comparison test. In addition, the associations of serum Hcy level with IVF/ICSI pregnancy outcomes were examined in infertile women with unexplained causes using Spearman correlation analysis and univariate and multivariate linear regression analyses. All statistical analyses were conducted using the statistical software SPSS version 19.0 (SPSS, Inc), and a *P* value < .05 was considered statistically significant.

## 3 RESULTS

### 3.1 Subjects characteristics

There were 129 infertile women in the case group and 259 infertile women in the control group. Table 1 shows the demographic and clinical features in the case and control groups. The age and body mass index (BMI) were both balanced between the two groups (*P* > .05); however, a significantly higher serum Hcy level was measured in the case group than in the control group (*P* = .008), and there was a significant reduction in the serum E_2_ level on the hCG day, fertilization rate, high‐quality embryo rate, total number of oocytes retrieved, and number of M‐II oocytes in the case group relative to the control group (*P* < .05).

**Table 1:** Comparison of baseline demographic and clinical features between groups

Characteristic	Case group (n = 129)	Control group (n = 259)	*P* value
Age (y)	31.07 ± 4.45	31.51 ± 4.41	.448
BMI (kg/m^2^)	21.73 ± 2.66	21.89 ± 2.57	.359
Day 3 FSH (IU/L)	6.65 ± 2.29	7.15 ± 3	.097
Antral follicle count	14.37 ± 3.55	13.54 ± 3.15	.462
AMH (ng/L)	4.53 ± 1.52	4.25 ± 1.58	.199
Serum Hcy level (μmol/L)	11 ± 4.46	7.12 ± 4.42	.008
Total gonadotropin dose used (IU/L)	2263.85 ± 874.58	2238.46 ± 811.29	.777
Duration of stimulation (d)	10.84 ± 2.34	10.62 ± 2.36	.389
Duration of infertility (y)	3.18 ± 2.26	3.55 ± 2.65	.183
Endometrial thickness on hCG day (mm)	10.14 ± 2.63	10.39 ± 2.47	.363
E_2_ level on hCG (pmol/L)	10 406.76 ± 3628.27	12 012.87 ± 3759.39	.014
TSH (IU/mL)	1.5 (1.5‐3.095)	1.7 (0.87‐3.1)	.363
anti‐Tg (IU/L)	3.2 (1.8‐6.7)	3.2 (1.7‐5.7)	.721
TpoA (IU/L)	1.4 (1.4‐3.4)	0.9 (0.4‐2.7)	.446
Type of infertility			
Primary infertility	77	127	.056
Secondary infertility	52	131	
Fertilization			
IVF	71	150	.561
ICSI	58	108	
Ovulation induction			
GnRH‐a long protocol	40 (31%)	95 (36.7%)	.66
GnRH‐ant protocol	42 (32.6%)	72 (27.8%)	
Super‐long protocol	12 (9.3%)	26 (10%)	
Modified long downregulation protocol	35 (27.1%)	66 (25.5%)	
Total number of oocytes retrieved	10 (7‐13.25)	12 (9‐17)	<.001
No. of M‐II oocytes	7 (5‐10)	9 (5‐12)	.006
Fertilization rate (%)	569 (60.6%)	1647 (69.4%)	<.001
High‐quality embryo rate (%)	357 (61.3%)	1090 (66.2%)	<.001

Abbreviations: AMH, anti‐müllerian hormone; anti‐Tg, anti‐thyroglobulin; BMI, body mass index; FSH, follicle‐stimulating hormone; ICSI, intracytoplasmic sperm injection; IVF, in vitro fertilization; TpoA, thyroid peroxidase antibody; TSH, thyroid‐stimulating Hormone.

### 3.2 Subgroup analysis of demographic features and IVF/ICSI outcomes in women with unexplained infertility stratified by serum Hcy

**Figure 1:**
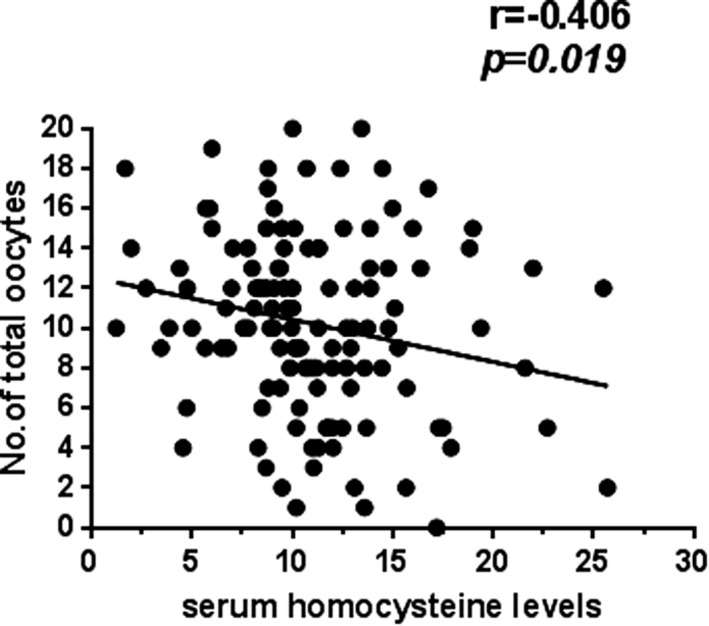
A negative correlation found between serum Hcy level and total number of oocytes retrieved

There was a significant difference in the total number of oocytes retrieved among subgroups A, B, and C (*P* = .031), and no significant difference was seen among these three subgroups in terms of age, BMI, E_2_ level on hCG day, number of M‐II oocytes, number of fertilized oocytes, or total number of high‐quality embryos (*P* > .05) (Table 2). Spearman correlation analysis revealed a negative correlation between serum Hcy level and total number of oocytes retrieved (*r* = −.406, *P* = .019) (Figure 1).

**Table 2:** Demographic and IVF/ICSI outcomes in women with unexplained infertility undergoing IVF/ICSI treatments stratified by serum Hcy level

Characteristics	Subgroup A (n = 26)	Subgroup B (n = 84)	Subgroup C (n = 19)
Age (y)	31.53 ± 4.41	31.12 ± 4.59	30.26 ± 3.89
BMI (kg/m^2^)	22.14 ± 3.87	21.64 ± 2.3	21.58 ± 2.2
E_2_ level on hCG day (pmol/L)	12 085.4 ± 5471	10 238 ± 5709.8	8853.4 ± 5158.2
Number of total oocytes retrieved	12.15 ± 3.8	9.95 ± 3.46	8.79 ± 4.07
Number of M‐II oocytes	8.81 ± 3.57	6.97 ± 3.86	6.68 ± 5.04
Number of fertilized oocytes	5.35 ± 2.58	4.25 ± 3.24	3.84 ± 2.7
Total number of high‐quality embryos	3.46 ± 2.38	2.52 ± 1.46	2.89 ± 1.18

### 3.3 Univariate and multivariate analyses of the correlation between serum Hcy level and IVF/ICSI outcomes

Since the aforementioned results showed a significant difference in the total number of oocytes retrieved among subgroups A, B, and C (*P* = .031), and a negative correlation between serum Hcy level and total number of oocytes retrieved (*r* = −.406, *P* = .019), we performed a univariate linear regression analysis to examine the association of serum Hcy level with total number of oocytes retrieved and number of M‐II oocytes, and a significant correlation was detected between serum Hcy level and total number of oocytes retrieved (Table 3). After adjusting for age, BMI, day 3 FSH, antral follicle count, AMH, serum Hcy level, total gonadotropin dose used, duration of stimulation, duration of infertility, endometrial thickness on hCG day, E_2_ level on hCG, TSH, anti‐Tg, TpoA, type of infertility, type of fertilization, type of ovulation induction, and infertility factors, the total number of oocytes retrieved was included in the multivariate linear regression model, and the multivariate linear regression analysis revealed that serum Hcy level had no correlations with any IVF/ICSI outcomes (Table 4).

**Table 3:** Univariate analysis of serum Hcy level with total number of oocytes retrieved and number of M‐II oocytes in women with unexplained infertility undergoing IVF/ICSI treatments

Univariate analysis	*B *(SE)	*t*	*P* value	95% CI	Tolerance	VIF
Total number of oocytes retrieved						
Serum Hcy level	−0.212 (0.088)	−2.4	.018	−0.387 to −0.037	1	1
Constant	12.561 (1.05)	11.963	<.001	10.483‐14.639		
Number of M‐II oocytes						
Serum Hcy level	−0.133 (0.079)	−1.676	.096	−0.29 to 0.024	1	1
Constant	8.745 (0.944)	9.269	<.001	6.878‐10.622		

**Table 4:** Multivariate analysis of factors associated with total number of oocytes retrieved in women with unexplained infertility undergoing IVF/ICSI treatments

Multivariate analysis	*B* (SE)	*t* Value	*P* value	95% CI	Tolerance	VIF
E_2_ level on hCG day	0.001 (0)	14.156	<.001	0.001‐0.001	0.994	1.006
Duration of infertility	−0.304 (0.108)	−2.81	−.517	−0.517 to −0.09	0.994	1.006
Constant	4.72 (0.638)	7.4	<.001	3.458‐5.983		

## 4 DISCUSSION

Although the exact pathogenesis of unexplained infertility has not been fully understood,^7^, ^8^, ^9^ hyperhomocysteinemia has been identified as a risk factor for unexplained infertility.^19^ Results from a case‐control study showed that lower Hcy levels in embryo culture medium were associated with a better chance of pregnancy and better embryo grades,^16^ and follicular fluid homocysteine levels were reported to be associated with clinical pregnancy, poor oocyte, and embryo qualities in polycystic ovary syndrome patients undergoing ART.^12^, ^17^ In addition, Hcy was reported to be inversely associated with fertility outcomes,^15^ and lower Hcy concentrations were measured in azoospermic seminal plasma than in normozoospermic seminal plasma.^20^ However, the association between Hcy level and unexplained infertility remains unclear until now. This retrospective, hospital‐based, case‐control study was therefore designed with aims to examine the association of Hcy with unexplained infertility in IVF/ICSI.

In this study, the case group exhibited a significantly higher serum Hcy level relative to the control group, and there were significant differences in the IVF/ICSI pregnancy outcomes between the case and control groups, including serum E_2_ level on the hCG day, fertilization rate, high‐quality embryo rate, total number of oocytes retrieved, and number of M‐II oocytes, which was in agreement with previous reports.^12^, ^16^, ^17^ Subgroup analysis revealed a significant difference in the total number of oocytes retrieved among subgroups A, B, and C, and Spearman correlation analysis and univariate linear regression analysis revealed a negative correlation between serum Hcy level and total number of oocytes retrieved; however, multivariate linear regression analysis revealed that serum Hcy level had no correlations with total number of oocytes retrieved, indicating that serum Hcy level had no associations with IVF/ICSI pregnancy outcomes.

Previous studies have shown that a high Hcy concentration may lead to a reduction in the number of oocytes, suppress oocyte maturity and fertilization, and reduce embryo quality.^21^ Elevated follicular fluid Hcy level may lead to follicular occlusion, which reduces the number and quality of oocytes and affects the quality of early embryos.^12^ High follicular fluid Hcy levels affect female reproductive functions, produce inflammatory cytokines, increase oxidative stresses, and disrupt the methylation reaction.^22^ All these changes are associated with oocyte development and fertilization, embryo implantation, and pregnancy maintenance.^21^, ^23^

It has been reported that that blood Hcy level reduces while E_2_ level on hCG day increases during ovarian stimulation.^24^ Although E_2_ was reported to regulate Hcy concentration,^22^ the underlying mechanisms are not understood. In order to decrease the negative effects of hyperhomocysteinemia, administration of high‐dose E_2_ and lowering Hcy level is thought to be beneficial in menopausal women. However, a higher E_2_ level on hCG day indicates more mature follicles retrieved and may increase the possibility of developing ovarian hyperstimulation.^25^ In the current study, there was a significant difference in the E_2_ level on hCG day between the case group and control group, which was in agreement with previous reports.^26^ It is therefore considered that Hcy may impact follicular growth and atresia, granular cell apoptosis, and hormone secretion, which plays an important role in the development of infertility.

The current study has some limitations. First, this is a single‐center, retrospective study. Second, the sample size seems relative small, notably in subgroup analysis. Further large, prospective, multi‐center studies are encouraged to validate the findings from this study.

In summary, the results of the present study demonstrate that serum Hcy level is greater in infertile women with unexplained causes than those with known causes; however, serum Hcy level has no associations with IVF/ICSI pregnancy outcomes, suggesting that serum Hcy level may be not a marker for the prediction of IVF/ICSI pregnancy outcomes. Further large‐scale prospective studies to examine the association between serum Hcy level and IVF/ICSI pregnancy outcomes seem justified.
